# Flow Injection Amperometric Measurement of Formalin in Seafood

**DOI:** 10.1021/acsomega.2c00515

**Published:** 2022-05-18

**Authors:** Kamonchanok Torrarit, Supatinee Kongkaew, Kritsada Samoson, Proespichaya Kanatharana, Panote Thavarungkul, Kah Haw Chang, Ahmad Fahmi Lim Abdullah, Warakorn Limbut

**Affiliations:** † Division of Health and Applied Sciences, Faculty of Science, Prince of Songkla University, Hat Yai, Songkhla 90110, Thailand; ‡ Forensic Science Innovation and Service Center, Prince of Songkla University, Hat Yai, Songkhla 90110, Thailand; § Center of Excellence for Trace Analysis and Biosensor, Prince of Songkla University, Hat Yai, Songkhla 90110, Thailand; ∥ Center of Excellence for Innovation in Chemistry, Faculty of Science, Prince of Songkla University, Hat Yai, Songkhla 90110, Thailand; ⊥ Division of Physical Science, Faculty of Science, Prince of Songkla University, Hat Yai, Songkhla 90110, Thailand; # Forensic Science Programme, School of Health Sciences, Universiti Sains Malaysia, Kubang Kerian 16150, Kelantan Malaysia

## Abstract

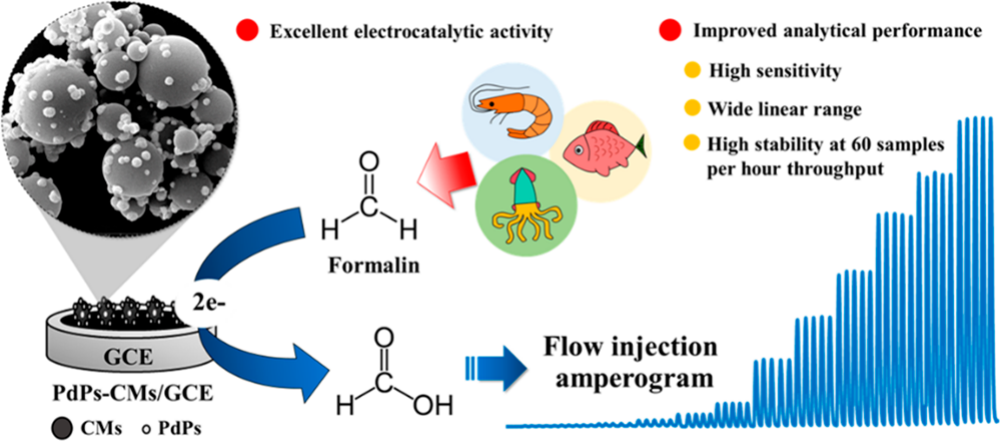

Formalin is illegally used as an antibacterial and a preservative in seafood products. It is extremely important for public health reasons to be able to simply, rapidly, and accurately detect formalin in fresh seafood. In this work, we developed a flow injection amperometric (FI-Amp) formalin sensor based on a glassy carbon electrode modified with a composite of palladium particles and carbon microspheres (PdPs-CMs/GCE). The CMs were decorated with PdPs via an electroless deposition method. The surface morphology of the CMs and the PdPs-CMs composite was characterized by scanning electron microscopy/energy dispersive X-ray spectroscopy (SEM/EDX). The electrochemical behavior and measurement of formalin at the PdPs-CMs/GCE was evaluated by cyclic voltammetry and amperometry. The modified electrode demonstrated good electrocatalytic performance for the oxidation of formalin. The synthesis method and FI-Amp operating conditions were optimized. Under the optimal conditions, the developed sensor showed a linear range of 0.025 to 15.00 mmol L^–1^ and a detection limit of 8 μmol L^–1^. Repeatability (RSD < 4.1%, *n* = 30), reproducibility (RSD = 0.25%, *n* = 5), stability (RSD = 3.2%, *n* = 80), and selectivity were good. The fabricated sensor achieved recoveries of formalin in seafood between 96 ± 1 to 105 ± 3 (*n* = 3).

## Introduction

Seafood is part of a healthy diet for most of the world’s population and is considered a rich source of high-quality protein, essential polyunsaturated fatty acids, minerals, and vitamins.^1^ The health benefits of eating seafood include childhood cognitive development, weight control, and reduced risks of certain cancers, coronary heart disease, stroke, high blood pressure, rheumatoid arthritis, and other inflammatory disorders.^2^ Consumption of seafood has increased in recent years as consumers have grown more aware of its nutritional benefits.^3^ The Food and Agriculture Organization (FAO) of the United Nations reported that the worldwide consumption of seafood increased from 9.9 kg per capita in 1960 to 19.7 kg per capita in 2013.^4^ The high demand for seafood products has led to an increased trade in seafood. However, the water, free amino acids, and fat in seafood make it susceptible to spoilage by microorganisms and biochemical reactions such as hypoxanthine and biogenic amine formation and changes in lipid and protein content.^5^ It is therefore essential to protect seafood from spoilage during storage and transport.

Unfortunately, the freshness of seafood is sometimes illegally preserved by the use of formalin, the liquid form of formaldehyde. Formalin has antimicrobial properties and can bind with proteins in seafood to cause muscle stiffness.^6^ However, the eaten seafood treated with formalin can cause abdominal pain, vomiting, coma, nephritic injury and risk of death.^7^ A million cases of food-borne illness have been reported from unsafe and contaminated foods.^8^ The contamination of fish, shrimp, and squid with formalin has been reported in several countries.^9^ The United States Environmental Protection Agency has set the reference dose of formalin at 0.2 mg/kg bodyweight/day.^10^ The International Agency for Research on Cancer (IARC) has classified formaldehyde as a Group 1 human carcinogen.^11^ In Thailand, formaldehyde is classified as a type 2 hazardous substance under the Hazardous Substance Act B.E. 2535 (C.E. 1992) and its use in food is prohibited under the Food Act B.E.2522 (C.E. 1979).^12^ Because of these health-related concerns, a rapid, reliable and highly sensitive sensor for the detection of formalin contamination in seafood is imperative.

Techniques that can be used to determine formalin include high-performance liquid chromatography,^13^ gas chromatography–mass spectrometry,^14^ spectrometry,^15^ chemiluminescence,^16^ and electrochemical sensors.^17,18^ The advantages of electrochemical sensors include fast response, simple operation, high sensitivity, good selectivity, on-site deployment, and effective detection without sample pretreatment.^17,19^ An amperometric approach is an attractive technique to producing an analytical signal, particularly when incorporated with a flow injection system (FI-Amp) for sample delivery. FI-Amp enables high throughput and rapid analysis with high reproducibility, good precision, and low sample contamination.^20,21^ For these reasons, FI-Amp was employed to enhance sensor performances in this work. However, the working electrode had to be modified with an electrocatalytic material to produce a more fully efficient electrochemical sensor.

Numerous transition metal particles were employed as catalytic materials to determine the target analyte in the food sample using an electrochemical sensor.^22^ Gold,^23,24^ platinum,^25^ and palladium^26,27^ particles have demonstrated high electrocatalytic activity derived from the d-orbit of surface atoms.^28^ Palladium particles (PdPs) in particular have exhibited their suitability for use in electrode modification since their electrocatalytic activity is high, toxicity is low, and stability is good.^27,29,30^ PdPs have also been developed with other materials into composites that exhibit enhanced electrocatalytic activity. Examples of PdPs composites are Pd-polymer,^31^ Pd-graphene (Pd-Gr),^32^ and Pd-carbon nanotubes (Pd-CNTs).^33^ These composites show better electrocatalytic performance than PdPs alone since they produce synergic effects. A composite of PdPs with carbon microspheres (CMs) is an interesting material that is simple to prepare. The properties of CMs include good particle size, size homogeneity, a wide potential window, high conductivity, and a large surface area that enables high metal particle loading.^34^ Furthermore, PdPs loadings on CMs have demonstrated effective distributions that increased catalytic activity, reduced electrode fouling, and accelerated electron transfer.^21,35^

Here, we proposed the electrocatalytic and synergistic properties of the PdPs-CMs composite for nonenzymatic formalin sensor. The cooperation of FI-Amp sensor provides a rapid and highly sensitive determination of formalin in seafood samples based on our composite. PdPs were synthesized on CMs through an electroless deposition method. The surface morphologies of a CMs/GCE and a PdPs-CMs/GCE were evaluated by scanning electron microscopy/energy dispersive X-ray spectroscopy (SEM/EDX), and the electrochemical properties of the electrodes were investigated by cyclic voltammetry (CV) and electrochemical impedance spectroscopy.^36^ The electrochemical response during formalin measurement was evaluated using the FI-Amp system. Critical operational parameters were evaluated, including the amount of PdPs-CMs composite in the drop casting solution, the applied potential, sodium hydroxide (NaOH) concentration, the carrier flow rate, and sample volume. Finally, the developed sensor was then applied to measure formalin in seafood samples.

## Experimentail Section

### Reagents and Materials

Palladium chloride (PdCl_2_), formalin (HCHO aqueous solution of 37% w/v formaldehyde), *N*, *N*-dimethylformamide (DMF), and Nafion were from Sigma-Aldrich (St. Louis, U.S.A.). Carbon microspheres powder was from SPI-Chem (West Chester, U.S.A.). Sodium borohydride and sodium hydroxide were from Merck KGaA (Darmstadt, Germany). Ultrapure water (18.2 MΩ cm) was utilized in the preparation of all chemical solutions.

### Instrumentation and Apparatus

The BiPotentiostat/Galvanostat μStat 400 (DropSens, Asturias, Spain) was employed in cyclic voltammetric and amperometric experiments and was controlled by Bluetooth signal through DropView 8400 software installed on a tablet computer. EIS studies were carried out with an AUTOLAB (Metrohm Autolab B.V., Utrecht, Netherlands) potentiostat-galvanostat with Nova 1.11 software. Scanning electron microscopy/energy dispersive X-ray spectroscopy (SEM/EDX, Quanta 400, FEI, U.S.A.) was used to characterize the surface morphology of the modified electrode.

#### Synthesis of PdPs-CMs Composite and Electrode Modification

**Figure 1:**
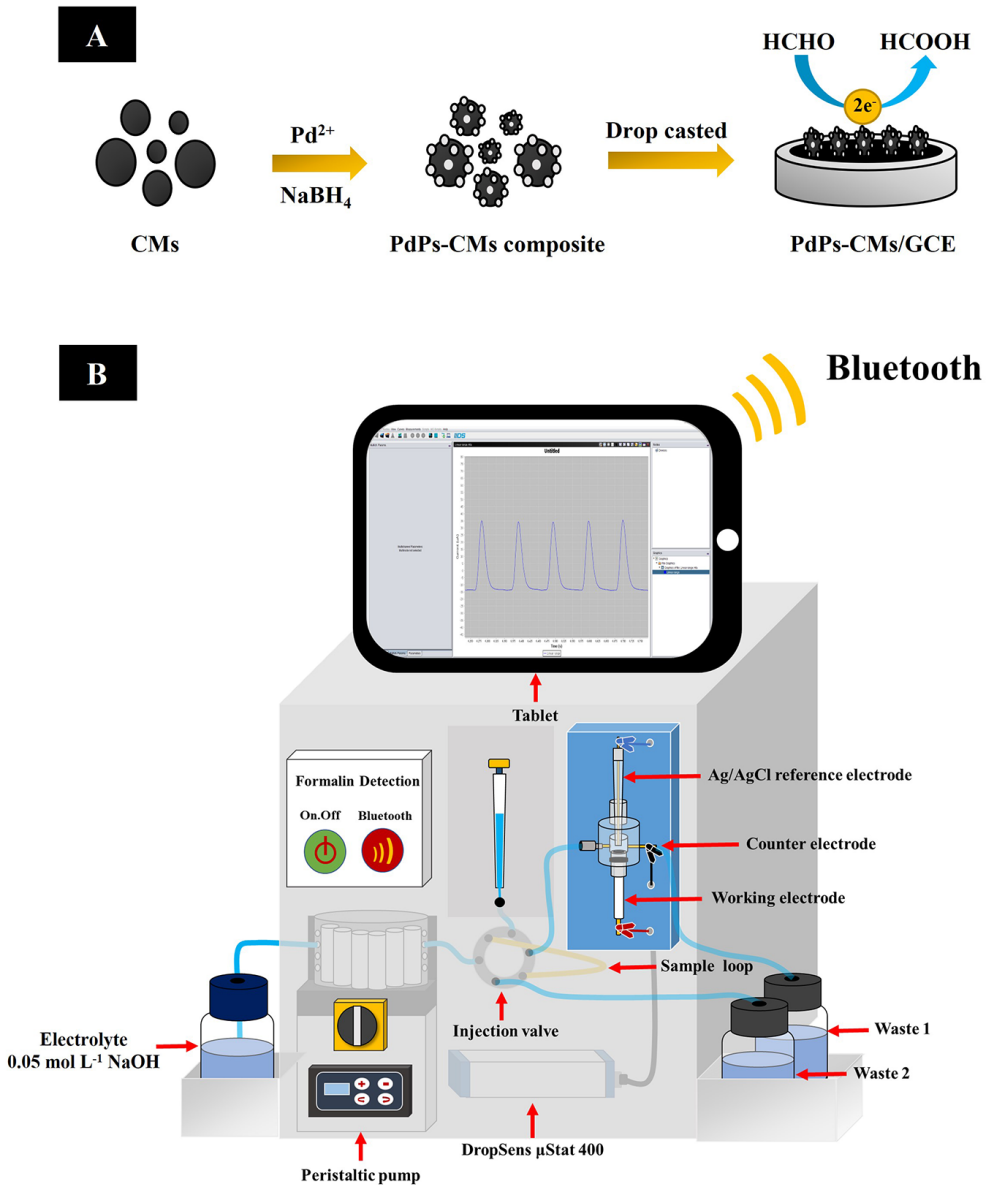
PdPs-CMs/GCE fabrication procedure (A). The schematic of the FI-Amp measurement system (B).

At room temperature, 0.2730 g of PdCl_2_ dissolved in 10 mL of 1.0 mol L^–1^ HCl was mixed with 100 mL of CMs suspension (2.0 mg mL^–1^ in water) under stirring. The pH of the mixture was adjusted to 10 with sodium hydroxide (1 mol L^–1^). The reducing agent, sodium borohydride (4.0 mg) was slowly added to the mixture under stirring to reduce palladium ions (Pd^2+^) to PdPs, which then adsorbed on the surface of CMs. The mixture was continuously stirred at room temperature for 24 h. After that, the obtained PdPs-CMs composite was filtered and washed with ethanol and ultrapure water several times until the pH of the waste water became neutral. The filtered composite was then dried overnight at 70 °C. For electrode modification, 2.0 mg mL^–1^ PdPs-CMs was dissolved in 1 mL of DMF under ultrasonication for 30 min. The Nafion binder was added to the mixture and continued to be sonicated until it became a homogeneous solution. After that, 15 μL of the PdPs-CMs (2 μL per time) was drop-casted onto the cleaned surface of the GCE (diameter = 3 mm) and dried at 70 °C to evaporate the solvent (Figure 1A).

### Electrochemical Characterization in a Batch System

In the batch system, the electrode array comprised a platinum wire act as a counter electrode, an Ag/AgCl (saturated in 3 mol L^–1^ KCl) act as a reference electrode, and the modified electrode act as a working electrode. To investigate electron transfer between the electrolyte and the fabricated electrode surface, a bare GCE, a CMs/GCE, and a PdPs-CMs/GCE, were electrochemically characterized in 5.0 mmol L^–1^ [Fe(CN)_6_]^4–/3–^ containing 0.10 mol L^–1^ KCl, using CV scanned from −0.40 to 1.20 V at a scan rate of 100 mV s^–1^. EIS experiment was studied in a frequency range of 100 kHz to 0.01 Hz at a frequency number of 50. The electrochemical behavior of formalin was studied by CV at the bare GCE, CMs/GCE, and PdPs-CMs/GCE, scanning from −0.90 to 0.80 V at a rate of 100 mV s^–1^ in 0.10 mol L^–1^ NaOH in the presence and absence of 1.00 mmol L^–1^ formalin. The influence of scan rate on the current response of formalin was also investigated in the same condition.

### FI-Amp Measurement

The portable FI-Amp system for the determination of formalin (Figure 1B) consisted of a six-port injection valve (Valco Instrument, U.S.A.), a peristaltic pump (Miniplus 3, Gilson, France), and a lab-built flow cell (dead volume 10 μL) containing a three-electrode system that was composed of the modified working electrode, a stainless-steel tube counter electrode, and an Ag/AgCl reference electrode. The system was connected to the DropSens μStat 400. The six-port injection valve was controlled the volume of sample and the peristaltic pump was employed to drive the carrier electrolyte of 0.10 mol L^–1^ NaOH. Formalin standard solutions were prepared in 0.10 mol L^–1^ NaOH and injected through the six-port injection valve. The amperometric signals of formalin oxidation were measured at a constant applied potential, and a constant sample volume and flow rate. The signals were transmitted via Bluetooth signal to the DropView 8400 software installed on a tablet computer.

### Sample Preparation and Analysis

Seafood samples including fish, shrimp, and squid were purchased from a local market in Hatyai, Songkla, Thailand and were prepared following a method previously described report.^37^ Briefly, small pieces of the seafood sample (20.0 g) were mixed with 100 mL of ultrapure water in a beaker. The mixture was shaken manually, left to stand for 2 h, and then passed through filter paper. The filtrate was analyzed using the developed sensor and the standard spectrophotometric method. For spectrophotometric detection, acetyl acetone reagent was mixed with the filtrate in a screw-cap glass vial and stored at normal room temperature for 2 h. The absorbance of the mixture was then determined by UV/vis spectrometry at a wavelength of 412 nm.^17^ A recovery test was also conducted by spiking the formalin standard solution concentration of 0.025, 0.050, 0.100, 0.250, and 0.500 mmol L^–1^ into the seafood samples.

## Results and Discussion

### Material Characterizations

**Figure 2:**
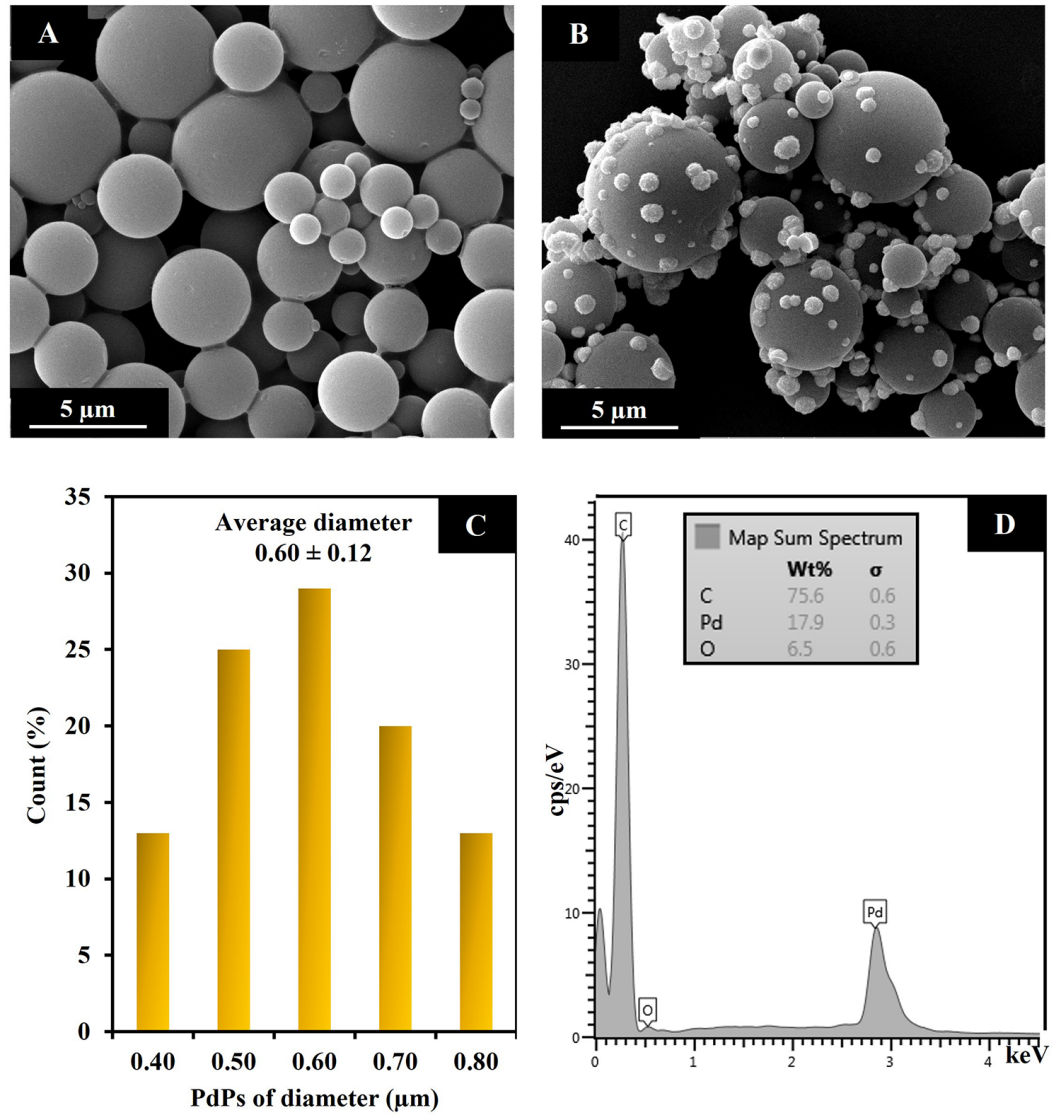
SEM images of CMs (A) and PdPs-CMs (B). Histogram of the size distribution of PdPs on CMs (C). EDX spectrum of PdPs-CMs (D).

The morphologies of the CMs and PdPs-CMs were characterized by SEM. The CMs exhibited smooth and clean surfaces. The CMs particle sizes ranged from 1 to 5 μm (Figure 2A), which provided a large surface area for decoration with PdPs. The PdPs-CMs, showed many small PdPs distributed on the surface of CMs (Figure 2B). The average diameter of the PdPs was 0.60 ± 0.12 μm (Figure 2C). The successful synthesis of the composite was confirmed by EDX analysis of PdPs-CMs, which produced a spectrum that showed the presence of C, Pd, and O (Figure 2D).

### Electrochemical Characterization of the Modified Electrode

**Figure 3:**
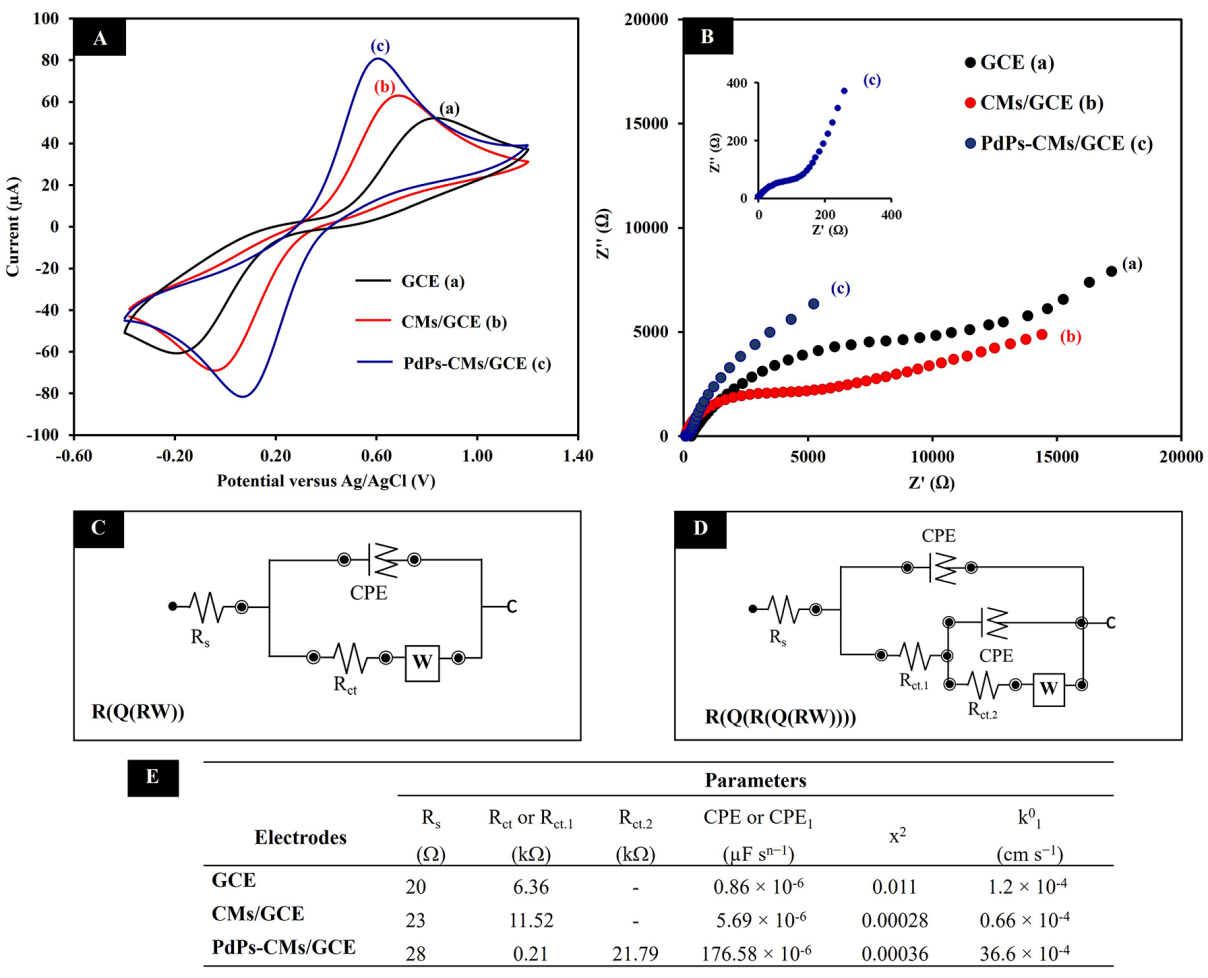
Cyclic voltammograms (A) and Nyquist plots (B) of GCE (a), CMs/GCE (b), and PdPs-CMs/GCE (c) in 5.0 mmol L^–1^ [Fe(CN)_6_]^4–/3–^ containing 0.10 mol L^–1^ KCl. Equivalent circuit for GCE, CMs/GCE (C) and PdPs-CMs/GCE (D). The summarized parameters values of the equivalent circuit from Nyquist plot obtained on GCE, CMs/GCE, and PdPs-CMs/GCE (E).

We used CV to investigate surface electron transfer at three different modified electrodes in a 0.10 mol L^–1^ KCl solution containing 5.0 mmol L^–1^ [Fe(CN)_6_]^4–/3–^. The CV curve produced at the bare GCE demonstrated well-defined anodic and cathodic peaks of [Fe(CN)_6_]^4–/3–^ with a peak-to-peak separation (Δ*E*_p_) of 1010 mV (Figure 3A(a)). The curve produced at the CMs/GCE (Figure 3A(b)) demonstrated the large surface area and high conductivity of CMs in the higher redox peak current and lower Δ*E*_p_ of 730 mV. The highest redox peak of the marker and lowest Δ*E*_p_ (690 mV) was produced at the PdPs-CMs/GCE (Figure 3A(c)). These results indicated that CMs and PdPs both enhanced conductivity and electron transfer at the electrode surface. To confirm the increased electrical conductivity of this electrode, EIS was employed. The semicircle diameter of the Nyquist plot impedance spectrum can be used to estimate charge transfer resistance (*R*_ct_). Randle’s equivalent circuit model *R*(*Q*(*RW*)) (Figure 3C) evaluated the *R*_ct.1_ values of the GCE (Figure 3B(a)) and CMs/GCE (Figure 3B(b)), which were 6.36 and 11.52 kΩ. The *R*_ct.1_ and *R*_ct.2_ values obtained from PdPs-CMs/GCE (Figure 3B(c)) were 0.21 and 21.79 kΩ fitted using Randle’s equivalent circuit model *R*(*Q*(*R*(*Q*(*RW*)))) (Figure 3D). These results were used to quantify the diffusion processes at the different electrodes by the heterogeneous electron-transfer-rate constant (*k*°) equation: *k*° = *RT*/(*n*^2^*F*^2^*AR*_ct_[*S*]). In this equation, *R*, *T*, *n*, *F*, *A*, *R*_ct_ and *S* refer to the universal gas constant, the temperature (K), the number of electrons, the Faraday constant, the geometric area of the electrode, the charge-transfer resistance, and the bulk concentration of the redox probe, respectively. The *k*°_1_ values calculated using *R*_ct.1_ were 1.2 × 10^–4^, 0.66 × 10^–4^, and 36.6 × 10^–4^ cm s^–1^ for the GCE, CMs/GCE, and PdPs-CMs/GCE, respectively. The *k*°_1_ value of the PdPs-CMs/GCE was thus 31-fold and 55-fold greater than the *k*° values of the GCE and CMs/GCE. More information obtained by fitting the semicircles of Nyquist plot impedance spectrum is shown in Figure 3E. The chi-square (χ^2^) values were small, indicating a high fit quality.^38^ These results indicate that the structures of PdPs/CMs could improve the bulk conductivity of the electrode surface to increase the active surface area and the rate of electron transfer.^39^

The electro-active surface areas of the different three kind electrodes were calculated using the Randles-Sevcik equation: *I*_p_ = 2.69 × 10^5^*An*^3/2^*D*^1/2^*C*υ^1/2^. For an electrolyte of 10 mM [Fe (CN)_6_]^3–/4–^, *n* = 1, *D* = 7.26 × 10^–6^ cm^2^ s ^–1^, and υ = 50 mV s^–1^. On the basis of data from the CV curves, the electroactive surface areas of the GCE, the CMs/GCE, and the PdPs-CMs/GCE were 0.07, 0.08, and 0.10 cm^2^, respectively. These results demonstrated that the PdPs-CMs composite material improved electrical conductivity and electron transfer at the electrode.

### Electrochemical Behavior of Formalin at the Modified Electrode

**Figure 4:**
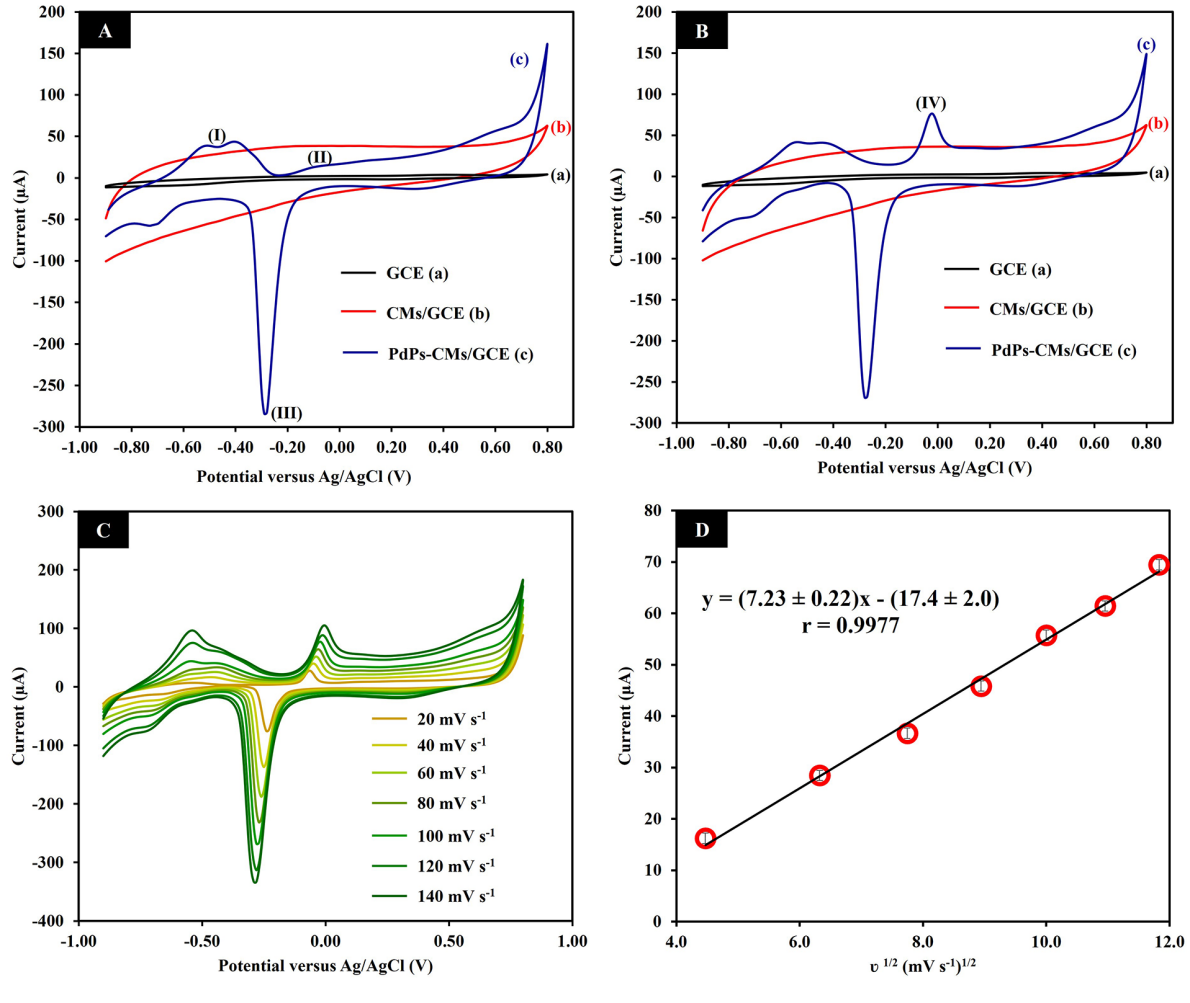
Cyclic voltammograms were produced at the GCE, CMs/GCE, and PdPs-CMs/GCE in 0.10 mol L^–1^ NaOH in the absence (A) and presence (B) of 1.00 mmol L^–1^ formalin scanned at 100 mV s^–1^. Cyclic voltammograms of the PdPs-CMs/GCE at scan rates from 20 to 140 mV s^–1^ in the presence of 1.00 mmol L^–1^ formalin (C). The relationship between the peak current of formalin oxidation versus the square root of the scan rate (D).

The electrocatalytic oxidation of formalin at the three electrodes was evaluated using CV in 0.10 mol L^–1^ NaOH. In the absence of formalin, the background current of the CMs/GCE (Figure 4A(b)) was larger than the background current of the GCE (Figure 4A(a)). This was likely due to the high conductivity and large surface area of CMs. After modification of the GCE with PdPs-CMs (Figure 4A(c)), three current peaks were observed at potentials of around −0.45, −0.10, and −0.30 V. The peaks corresponded to the desorption and oxidation of adsorbed hydrogen (peak I, reaction 1), the formation of Pd oxide (peak II, reactions 2, 3, and 4) and the reduction of Pd oxide (peak III, reaction 5)^40^
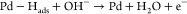
1
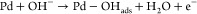
2
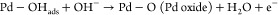
3
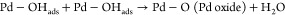
4
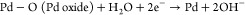
5In the presence of 1.00 mmol L^–1^ formalin, the GCE (Figure 4B(a)) and CMs/GCE (Figure 4B(b)) demonstrated no obvious electrochemical response toward formalin. This lack of response was due to the lack of catalytic activity between the two electrodes and formalin. However, at the PdPs-CMs/GCE, a well-defined oxidation peak of formalin was clearly observed at a potential of about 0 V (Figure 4B(c)). In alkaline media, methylene glycol (H_2_C(OH)_2_), which is the active intermediate of formalin oxidation, is produced by the reaction between formalin (HCHO) and water (H_2_O). At the PdPs-CMs/GCE, the intermediate is oxidized to produce formic acid by the catalytic activity of Pd–O (peak IV, reaction 6). The electrochemical oxidation mechanism of formalin in the presence of Pd has been previously described.^26,29^
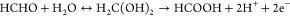
6To evaluate the electrochemical kinetics of formalin oxidation on the electrode surface, the influence of scan rate on the anodic peak current of formalin was investigated using CV, scanning from 20 to 140 mV s^–1^ in 0.10 mol L^–1^ NaOH containing 1.00 mmol L^–1^ formalin (Figure 4C). The plot between the anodic current response and the square root of the scan rate provided good linearity with a linear regression equation of *I*_pa_ (μA) = (7.23 ± 0.22)v^1/2^ (mV s^–1^) – (17.4 ± 2.0) (μA) (*r* = 0.9977) (Figure 4D). The good linear relationship demonstrates that the oxidation of formalin at the PdPs-CMs/GCE was a diffusion-controlled process.^27,41^

### Formalin Sensor Optimization

In order to obtain the best electrocatalytic detection of formalin at the PdPs-CMs/GCE in the FI-Amp system, we optimized the amount of PdPs-CMs on the electrode surface, the potential applied at the working electrode, NaOH concentration, flow rate, and sample volume. The initial operating conditions for the FI-Amp system were −0.10 V of applied potential, 0.10 mol L^–1^ of NaOH, 1.00 mL min^–1^ of flow rate, and 250 μL of sample volume. All parameters were tested with standards of 0.25, 0.50, 1.00, 5.00, and 10.00 mmol L^–1^ formalin. The condition that produced the highest sensitivity was considered optimal.

#### Amount of PdPs-CMs on GCE

**Figure 5:**
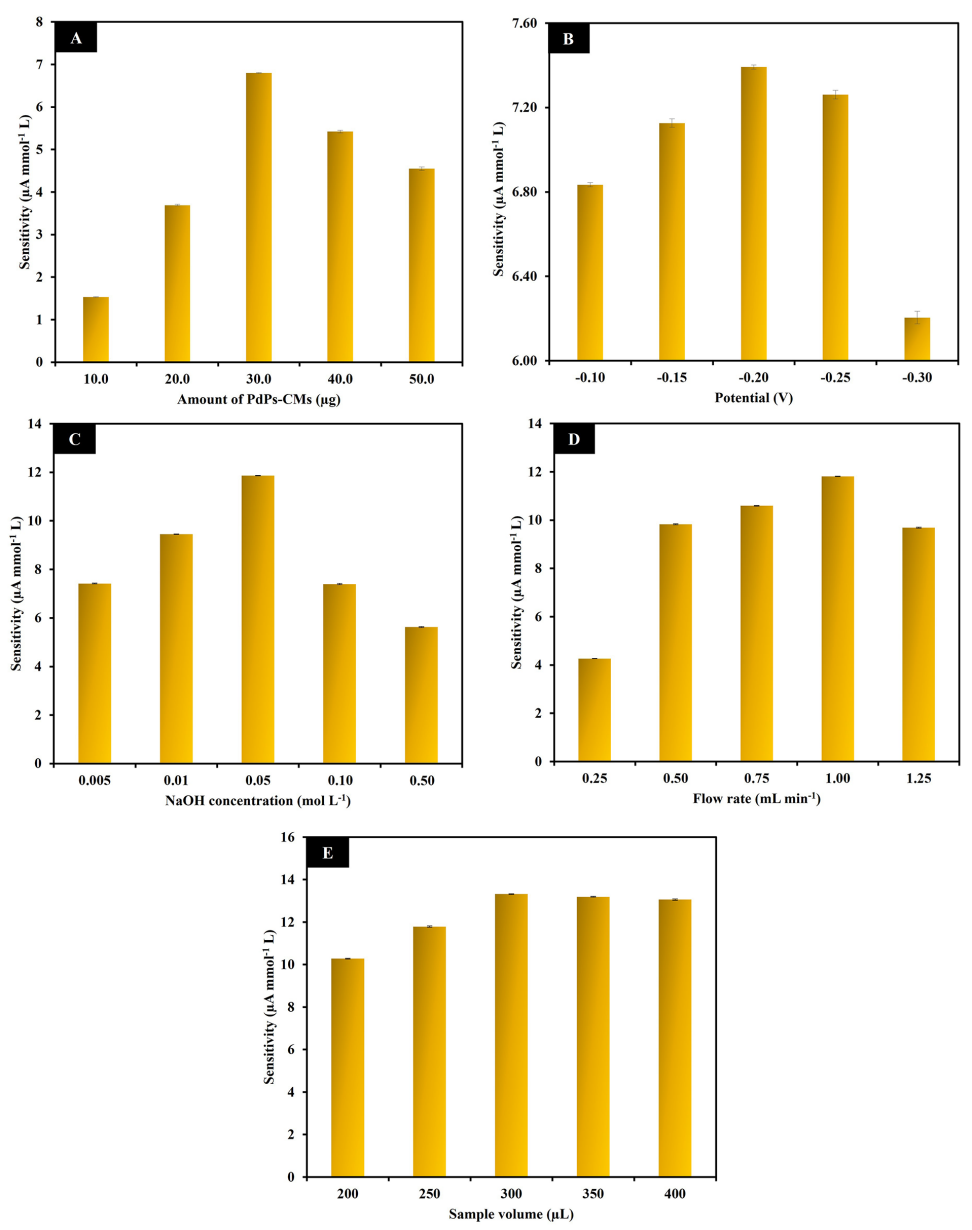
Charts show the effect of the amount of PdPs-CMs composite in the drop casting solution (A), the applied potential (B), the NaOH concentration (C), the flow rate (D) and the sample volume (E) on the sensitivity of formalin measurement at the PdPs-CMs/GCE. Initial conditions were −0.10 V applied potential, 0.10 mol L^–1^ NaOH, 1.00 mL min^–1^ flow rate, and 250 μL sample volume. Formalin concentrations were determined at 0.25, 0.50, 1.00, 5.00, and 10.00 mmol L^–1^.

The electrocatalytic detection of formalin by the flow-based electrochemical sensor is strongly influenced by the PdPs-CMs loading on the GCE. The GCE was modified with PdPs-CMs loadings of 10.0, 20.0, 30.0, 40.0, and 50.0 μg. The sensitivity of the current response initially increased as the loading of PdPs-CMs increased from 10.0 to 30.0 μg and then gradually decreased (Figure 5A). The increase in sensitivity was due to the increasing amount of catalyst on the GCE surface.^17^ The lower sensitivity to PdPs-CMs loads greater than 30.0 μg probably occurred because the thickness of the PdPs-CMs composite on the GCE surface hindered electron transfer.^17,42^ Therefore, 30.0 μg of PdPs-CMs composite was employed for electrode modification.

#### Formalin Oxidation Potential

The potential applied to the working electrode is particularly important for the production of a selective and sensitive amperometric signal. A series of potentials were applied that covered the oxidation peak in the cyclic voltammogram produced at the PdPs-CMs/GCE by formalin (Figure 4B). The applied potentials were −0.10, −0.15, −0.20, −0.25, and −0.30 V. Sensitivity increased with increments of applied potential up to −0.20 V and then decreased above −0.20 V (Figure 5B). Therefore, −0.20 V was chosen as the appropriate potential.

#### NaOH Concentration

The concentration of NaOH was an important factor affecting the electrocatalytic behavior of formalin at the PdPs-CMs/GCE since it caused the formation of the active intermediate of methylene glycol and the Pd–O that catalyzed formalin oxidation.^43^ The effect of NaOH concentration on the sensitivity of response was studied at 0.005, 0.01, 0.05, 0.10, and 0.50 mol L^–1^. The current response demonstrated the highest sensitivity at 0.05 mol L^–1^ (Figure 5C). At concentrations below 0.05 mol L^–1^, there were probably not enough hydroxide ions (OH^–^) to produce sufficient methylene glycol and Pd–O, and at higher concentrations there were enough hydroxide ions to occupy the active sites of the electrode surface.^44^ Therefore, a NaOH concentration of 0.05 mol L^–1^ was chosen for further studies.

#### Flow Rate and Sample Volume

Flow rate and sample volume are significant parameters in flow injection analysis since the contact time between analyte and electrode surface can affect current response and analysis time. To obtain the optimal electrocatalytic behavior of formalin at the PdPs-CMs/GCE in the FI-Amp system, the flow rate was varied from 0.25–1.25 mL min^–1^ at increments of 0.25 mL min^–1^. A flow rate of 1.00 mL min^–1^ produced the highest sensitivity (Figure 5D). At lower flow rates, mass transfer between formalin and the surface of the electrode was probably low, and at the higher flow rate, the contact time between formalin and the PdPs-CMs/GCE was probably too short.^17,45^ The sample volume was varied from 200 to 400 μL at increments of 50 μL. The sensitivity of the amperometric response increased in response to increments of sample volume up to 300 μL and then remained almost constant (Figure 5E). As the sample volume increased, the contact time between formalin and the PdPs-CMs/GCE surface also increased, which enhanced electrocatalytic activity and therefore the sensitivity of the electrode. However, at sample volumes higher than 300 μL, contact time was longer, sensitivity stabilized, but analysis time increased.^45,46^ To achieve the highest sensitivity and a short analysis time, a flow rate of 1.00 mL min^–1^ and a sample volume of 300 μL were selected.

### Analytical Performance

#### Linearity and Limit of Detection

**Figure 6:**
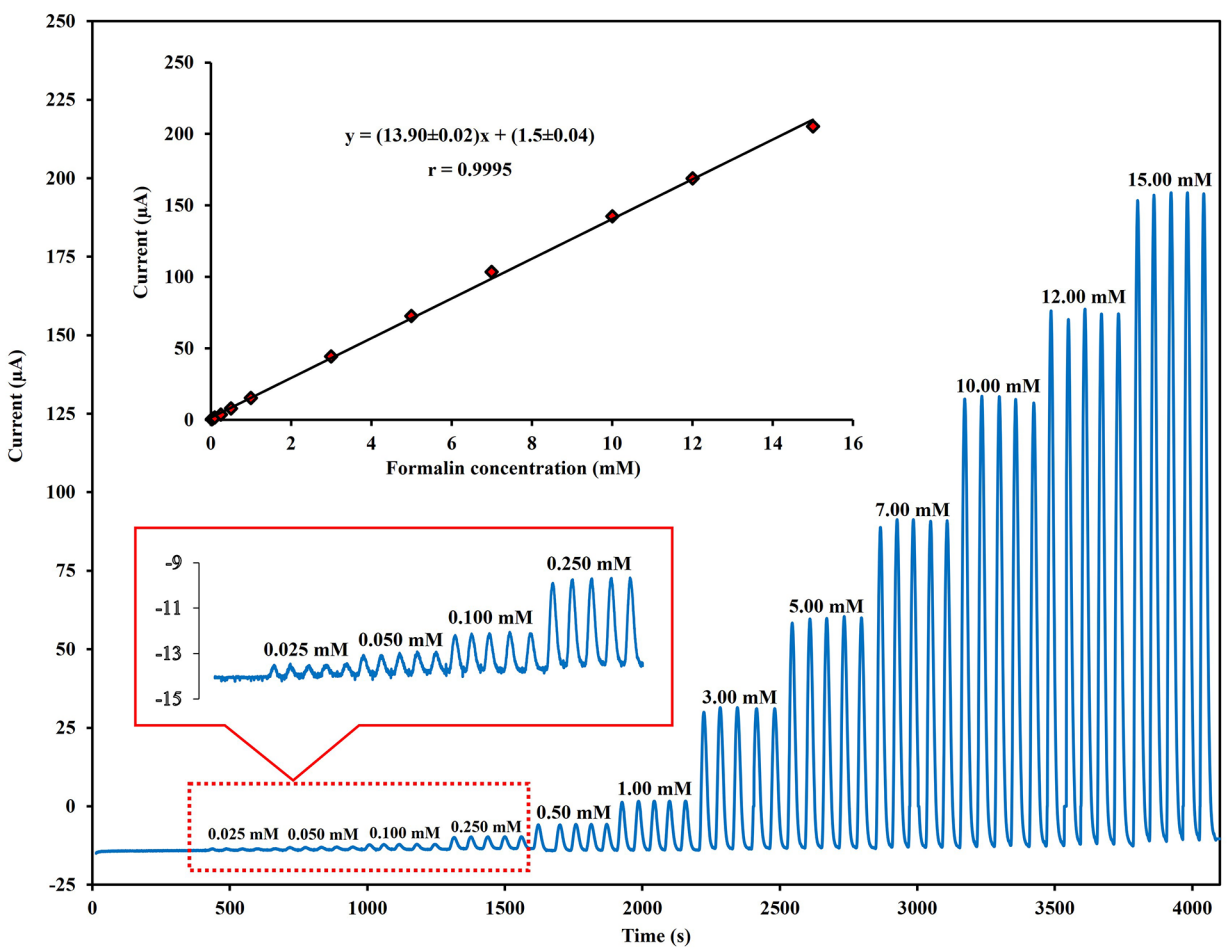
Flow injection amperometric responses to different concentrations of formalin (0.025 to 15.00 mmol L^–1^) were measured at the PdPs-CMs/GCE in the optimal condition. Inset; calibration plot of the amperometric signal versus formalin concentration.

The analytical performance of the flow-based electrochemical formalin sensor was investigated in the optimal conditions. The amperometric response increased with increments of formalin concentration (Figure 6). A good linear relationship existed between the current response and concentration from 0.025 to 15.00 mmol L^–1^ (*r* = 0.9995). The limit of detection (LOD) was 8 μmol L^–1^. The calculation was based on the equation LOD = 3σ/S, where σ is the standard deviation of the blank (*n* = 20) and *S* is the slope of the calibration curve (Figure 6 (inset)). When compared with other electroanalytical methods for the measurement of formalin (Table 1), the proposed sensor exhibited a wide linear range and the lowest detection limit. The good analytical performances of the FI-Amp system may be attributed to the combination of good electrocatalytic activity and the synergistic properties of PdPs-decorated CMs.

**Table 1:** Comparison of Analytical Performances of the Developed Formalin Sensor and Other Electroanalytical Methods for the Measurement of Formalin

modified electrode	technique	linear range (mmol L^–1^)	LOD (μmol L^–1^)	sample	reference
aAuNPs/PPy/GCE	hDPV	0.4–2.4	20	milk	(23)
bPdNPs-PAA-GO/GCE	iFI-Amp	0.05–50	16	fresh food	(17)
cPt/EG/GCE	jCV	0.125–2.00	40		(47)
dCdS/Chitosan/PtE	CV	0.167–1.67	166	fish	(48)
ePd/TiO_2_ electrode	kAmp	0–17.7	15		(26)
fNanoPd electrode	amp	0–20	38		(49)
gPdPs-CMs/GCE	FI-amp	0.025–15	8	seafood	this work

a AuNPs/PPy/GCE: gold nanoparticles-polypyrrole composite modified glassy carbon electrode.

b PdNPs-PAA-GO/GCE: palladium nanoparticle-poly(acrylic acid)-graphene oxide modified on a glassy carbon electrode.

c Pt/EG/GCE: platinum-electrochemically reduced graphene modified glassy carbon electrode.

d CdS/Chitosan/PtE: cadmium sulfide nanoparticles-chitosan modified glassy carbon electrode.

e Pd/TiO_2_ electrode: palladium–titanium dioxide electrode.

f NanoPd electrode: nanopalladium electrode.

g PdPs-CMs/GCE: palladium particles-carbon microspheres composite modified glassy carbon electrode.

h DPV: differential pulse voltammetry.

i FI-Amp: flow injection amperometry.

j CV: cyclic voltammetry.

k Amp: amperometry.

#### Repeatability, Reproducibility, and Stability

**Figure 7:**
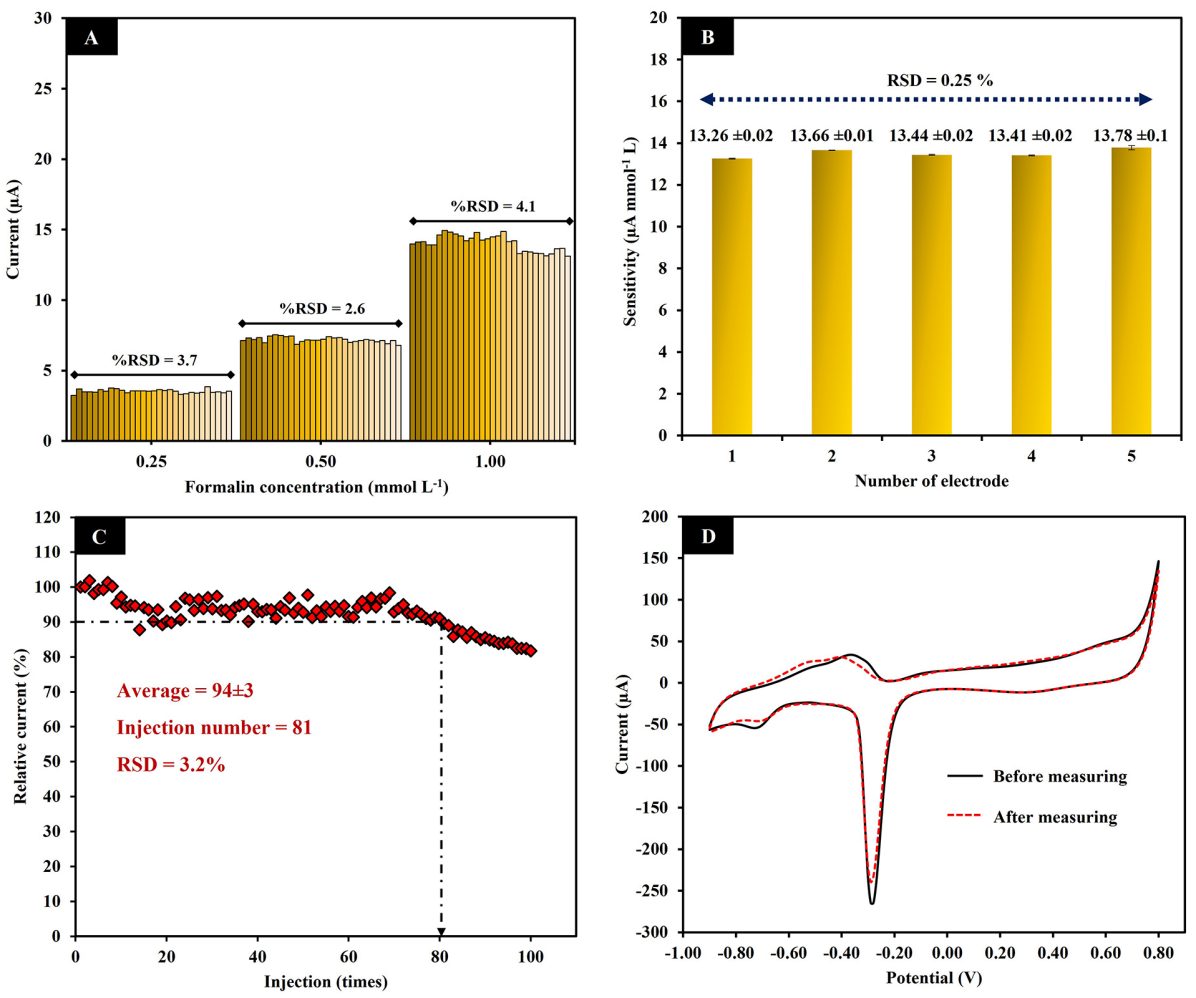
Current responses at the PdPs-CMs/GCE to 30 injections of three concentrations of formalin (0.25, 0.50, 1.00 mmol L^–1^) (A). The sensitivity of five PdPs-CMs/GCE preparations (B). Operational stability of the PdPs-CMs/GCE (D). Cyclic voltammograms produced before and after measuring formalin (C).

The repeatability of the PdPs-CMs/GCE was evaluated by measuring the flow injection amperometric responses of 30 injections of standard formalin solution at 0.25, 0.50, and 1.00 mmol L^–1^. The relative standard deviations (RSDs) of the 15 measurements were 3.7%, 2.6%, and 4.1% for 0.25, 0.50, and 1.00 mmol L^–1^, respectively (Figure 7A). These RSD values are acceptable results according to the AOAC recommendations^50^ and show the excellent repeatability of the developed method. To investigate the reproducibility of the electrode, five PdPs-CMs/GCEs were prepared at different times to measure formalin concentrations of 0.25, 0.50, 1.00, 5.00, and 10.00 mmol L^–1^. The RSD of the average sensitivity of the five electrode preparations was 0.25% (Figure 7B). This result indicates the good reproducibility of the preparation of the PdPs-CMs/GCE. The operational stability of PdPs-CMs/GCE was studied by measuring the amperometric signal in response to repeated injections of 1.00 mmol L^–1^ formalin at a throughput of 60 samples h^–1^ (Figure 7C). The PdPs-CMs/GCE demonstrated good stability for 80 injections, producing an average amperometric signal of 94 ± 3% of the initial signal. The RSD was 3.2%. After 80 injections, the amperometric signal fell below 90% of the initial signal. The decrease in the signal was probably due to the loss of PdPs-CMs composite, which would reduce the number of catalytic sites available to the analyte. The loss of catalytic material was confirmed by CV. The cyclic voltammogram (Figure 7D) shows the decrease in the cathodic peak of Pd after 80 formalin injections.

#### Interference Study

The PdPs-CMs/GCE was applied to measure formalin in the presence of interfering species that may exist in real-world seafood samples. The interferences were Ca^2+^, Na^+^, NH_4_^+^, K^+^, CO_3_^2–^, SO_4_^2–^, Cl^–^, NO_3_^–^, and PO_4_^3–^. The tolerance limit for each interfering substance was defined as the maximum concentration that produced a relative error of less than ±5.0% in the determination of formalin at a concentration of 1.00 mmol L^–1^. The signal was not significantly affected in the presence of 25-fold Ca^2+^, CO_3_^2–^, Cl^–^, and PO_4_^3–^, 50-fold NH_4_^+^, K^+^ and NO_3_^–^ and 100-fold Na^+^ and SO_4_^2–^ (Table 2). All relative errors were below 5%, and thus there was no significant interference effect. These results exhibited that the PdPs-CMs/GCE was selective toward formalin.

**Table 2:** Tolerance Ratios of Various Interferences Present during the Measurement of 1.00 mmol L^–1^ Formalin by the PdPs-CMs/GCE

interference species	tolerance ratio [interference]/[1.00 mmol L^–1^ formalin]	relative error (%)
Ca^2+^	25	±3
Na^+^	100	±1
NH_4_^+^	50	±1
K^+^	50	±2
CO_3_^2–^	25	±3
SO_4_^2–^	100	±1
Cl^–^	25	±1
NO_3–_	50	±2
PO_4_^3–^	25	±2

#### Analysis of Seafood Samples

To evaluate its practical application, the developed sensor was applied in the optimum condition to detect formalin in seafood samples. Six seafood samples were prepared as described in the sample preparation and analysis section and then measured with the developed sensor and the standard spectrophotometric technique.

The concentration of formalin detected in the real samples could not be determined using either method because it was below the LOD value of both (proposed method = 8 μmol L^–1^, spectrophotometric method = 7.50 μmol L^–1^). Following that, formalin concentrations of 0.025, 0.050, 0.100, 0.250, and 0.500 mmol L^–1^ were added to six seafood samples. Recoveries were obtained from all six seafood samples at all five spiked concentrations. The obtained recovery values ranged from 96 ± 1 to 105 ± 3 for the fabricated sensor and 92 ± 2 to 108 ± 4 for the spectrophotometric technique (Table 3). These values are well within the limits of the AOAC guideline,^50^ therefore this sensor can accurately and reliably determine formalin in seafood samples.

**Table 3:** Formalin Measurement in Seafood Samples (*n* = 3) and the Recoveries

		% recovery of spectrometry method (*n* = 3) concentration of spiking (mmol L^–1^)						% recovery of proposed method (*n* = 3) concentration of spiking (mmol L^–1^)				
sample	spectrometric method	0.025	0.050	0.100	0.250	0.500	proposed method	0.025	0.050	0.100	0.250	0.500
splendid squid	ND	104 ± 6	101 ± 4	99 ± 2	103 ± 5	99. ± 5	ND	102 ± 2	100 ± 4	105 ± 3	103 ± 1	102 ± 3
dollfus’ octopus	ND	108 ± 4	100 ± 1	94 ± 4	103.8 ± 0.4	102 ± 5	ND	100 ± 5	98 ± 2	102 ± 1	102 ± 4	102 ± 1
rainbow cuttlefish	ND	102 ± 4	100 ± 1	96 ± 3	103 ± 1	97 ± 5	ND	100 ± 4	100 ± 2	102 ± 2	101 ± 3	100 ± 2
Pacific white shrimp	ND	104 ± 4	100 ± 2	98 ± 3	102 ± 2	101 ± 5	ND	99 ± 2	104 ± 1	101 ± 5	96 ± 1	101.5 ± 0.9
mackerel	ND	102.6 ± 0.4	98 ± 2	96 ± 1	99 ± 1	101.8 ± 0.5	ND	100 ± 1	103 ± 2	103 ± 1	102 ± 2	99.8 ± 0.8
torpedo scad	ND	103 ± 1	98 ± 1	92 ± 2	104 ± 1	103 ± 4	ND	102 ± 2	102 ± 2	100 ± 4	102 ± 4	99 ± 1

## Conclusions

A flow-based electrochemical formalin sensor was successfully developed based on a glassy carbon electrode modified with a composite of palladium particles and carbon microspheres. The composite exhibited good electrocatalytic activity and synergistic properties that enhanced the analytical performances of the sensor. Under optimal conditions, the proposed formalin sensor produced an amperometric signal with a low detection limit (8 μmol L^–1^), high sensitivity, and wide linear range (0.025 to 15.00 mmol L^–1^). The sensor also demonstrated good repeatability, reproducibility, and stability at a throughput of 60 samples per hour. In addition, the interfering substances that may exist in seafood had no effect on formalin detection. The developed sensor measured formalin in seafood samples with high accuracy.
